# Weighted Gene Co-Expression Analysis Network-Based Analysis on the Candidate Pathways and Hub Genes in Eggplant Bacterial Wilt-Resistance: A Plant Research Study

**DOI:** 10.3390/ijms222413279

**Published:** 2021-12-10

**Authors:** Jiechun Peng, Peng Wang, Huarong Fang, Jieming Zheng, Chuan Zhong, Yanjuan Yang, Wenjin Yu

**Affiliations:** College of Agriculture, Guangxi University, Nanning 530004, China; 1917302008@st.gxu.edu.cn (J.P.); wangpeng@gxaas.net (P.W.); 2017302001@st.gxu.edu.cn (H.F.); 2017302011@st.gxu.edu.cn (J.Z.); zc@gxu.edu.cn (C.Z.); yjyang85@126.com (Y.Y.)

**Keywords:** eggplant, bacterial wilt-resistance, weighted gene co-expression analysis network, pathways, hub genes, VIGS

## Abstract

*Solanum melongena* L. (eggplant) bacterial wilt is a severe soil borne disease. Here, this study aimed to explore the regulation mechanism of eggplant bacterial wilt-resistance by transcriptomics with weighted gene co-expression analysis network (WGCNA). The different expression genes (DEGs) of roots and stems were divided into 21 modules. The module of interest (root: indianred4, stem: coral3) with the highest correlation with the target traits was selected to elucidate resistance genes and pathways. The selected module of roots and stems co-enriched the pathways of MAPK signalling pathway, plant pathogen interaction, and glutathione metabolism. Each top 30 hub genes of the roots and stems co-enriched a large number of receptor kinase genes. A total of 14 interesting resistance-related genes were selected and verified with quantitative polymerase chain reaction (qPCR). The qPCR results were consistent with those of WGCNA. The hub gene of *EGP00814* (namely *SmRPP13L4*) was further functionally verified; *SmRPP13L4* positively regulated the resistance of eggplant to bacterial wilt by qPCR and virus-induced gene silencing (VIGS). Our study provides a reference for the interaction between eggplants and bacterial wilt and the breeding of broad-spectrum and specific eggplant varieties that are bacterial wilt-resistant.

## 1. Introduction

*Solanum melongena* L. (eggplant) bacterial wilt is a devastating soil borne disease caused by *Ralstonia solanacearum* that severely restricts the growth, yield, and production of eggplants. China produces the highest amount of eggplants, which are widely cultivated throughout the country. In China, the main pathogen of eggplant bacterial wilt, *R. solanacearum*, often occurs south of the Yangtze River wherein eggplant production is restricted by 20–30% in seasons of mild infection with low pathogen incidence and by 80–100% in seasons of severe infection [1,2].

Understanding the mode of *R. solanacearum* infection, its pathogenic mechanism, and the *R. solanacearum* resistance mechanism plays an indispensable role in the control of *R. solanacearum*. *R. solanacearum* mainly invades natural orifices and wounds of roots before rapidly proliferating in the xylem and spreading to the stem. During this process, the pathogen induces change in the walls and membranes of the cells, deposits callose, produces lipopolysaccharide, and blocks catheters, thereby leading to plant wilting. When *R. solanacearum* infects plant cells, the cells’ pattern recognition receptors (PRRs) respond to pathogen/microbe-associated molecular patterns in the P/MAMPs mode, which stimulates ion flux changes, produces reactive oxygen species, and triggers the first anti-epidemic pattern-triggered immunity (PTI) of cells. When the pathogen overcomes the PTI, it enters the cells. The nucleotide-binding leucine-rich repeat (NLR) protein family of intracellular immune receptors then binds to the pathogen and triggers the second anti-epidemic effector-triggered immunity (ETI) of plant cells. This is accompanied by hypersensitivity reaction (HR), changes in defence-related gene expression, production of mitogen-activated protein phosphorylated kinase (MAPK) and plant hormone [3,4,5], and induction of systemic acquired resistance (SAR). After infection by *R. solanacearum*, salicylic acid (SA) and ethylene (ET) play a major role in plant hormones. SA marker genes, such as *PAD4*, *NPR1*, *SGT1*, *TGA*, *Glua*, and *PR-1a*, were upregulated in eggplants, while ET marker genes, such as *OSM* and *PR-1b*, were upregulated in tomatoes and potatoes [6,7]. However, the *SmNAC* gene inhibits bacterial wilt-resistance by inhibiting the expression of heterocladate synthase-1 to decrease the content of SA [8]. Hamilton et al. [9] used infected tomato plants in bacterial GMI1000 and its mutants to reveal that inositol was used as the carbon source of roots in the early stage, while sucrose and trehalose were used as carbon sources of stems. The interaction between eggplants and *R. solanacearum* was extremely complex. To further interpret the regulatory mechanism in eggplant bacterial wilt-resistance and screen the varieties with broad-spectrum resistance to bacterial wilt more rapidly, gene mapping and transcriptome sequencing have been widely adopted.

With the rapid development of next generation sequencing technology (NGS), RNA-seq has been employed as a simple method for identifying resistance genes at the genome level through the analysis of transcriptional maps generated throughout the genome. Previous studies on Solanaceae crops inoculated with *R. solanacearum* showed that in eggplants, auxin sensitive inhibitor *Aux/IAA* gene, JA pathway *MYC2* and *JAZ* genes, SA pathway *PR1* gene, and transcription factor *WRKY* family *SiWRKY53* were ideal target genes for the molecular breeding of eggplants with bacterial wilt-resistance [10,11]. Li et al. [12] revealed that *WRKY6* and *WRKY11* genes of the *WRKY* family, *ERF5* and *ERF15* genes of the *ERF* family, and *PR5* genes were significantly upregulated in tobacco. Cao et al. [13] revealed that the most significant specific pathway of bacterial wilt infection in potatoes was the transformation of pentose and glucuronic acid. This pathway played an important role in the formation of plant cell walls [13]. In addition, Alonso-Díaz et al. [14] revealed that cytokinins played an important role in the early root defence of *Arabidopsis* plants infected with GMI1000. The mechanism of control of Solanaceae crops against bacterial wilt has been gradually elucidated. However, there remain unsolved problems that require further study. 

Co-expression network analysis has been widely used in bioinformatics. Based on conventional analysis of the transcriptome, co-expression network analysis can identify highly correlated gene subsets within the network, provide new insights on the role of different genes related to specific conditions, and serve as an important tool for identifying gene co-expression associated with phenotype. For example, weighted gene co-expression network analysis (WGCNA) is a systems biology method used to describe the correlation patterns between genes in microarray samples. WGCNA can find clusters, also known as modules, of highly related genes. Module characteristic genes or key genes in modules summarise these clusters and associate them with samples or traits to obtain candidate key genes and metabolic pathways [15,16,17,18]. Based on WGCNA, Zhu et al. [19] predicted six salt-responsive core hub genes and several key transcription factors in rice. Yuan et al. [20] mined 15 gene co-expression modules closely related to root drought resistance of potatoes and 20 core genes from four modules with the highest correlation with the target traits. These results can be used for further studies on the molecular genetic mechanisms of rice and potatoes.

In the present study, we used scanning electron microscopy (SEM) to observe *R. solanacearum* infection in eggplants in vivo. Based on RNA-seq, the transcriptome of the roots and stems of BC01 and Rf were sequenced in response to *R. solanacearum* infection in four stages: uninoculated, 1 day post-inoculation, early disease onset, and peak disease onset. To explore candidate pathways and hub genes for bacterial wilt-resistance, a transcriptome sequencing analysis with WGCNA, qPCR, and VIGS were conducted. The results of this study have important theoretical significance for elucidating the regulation mechanism of bacterial wilt-resistance in eggplants and for breeding broad-spectrum and specific resistant varieties.

## 2. Results

### 2.1. Phenotypic and Histological Observation

**Figure 1:**
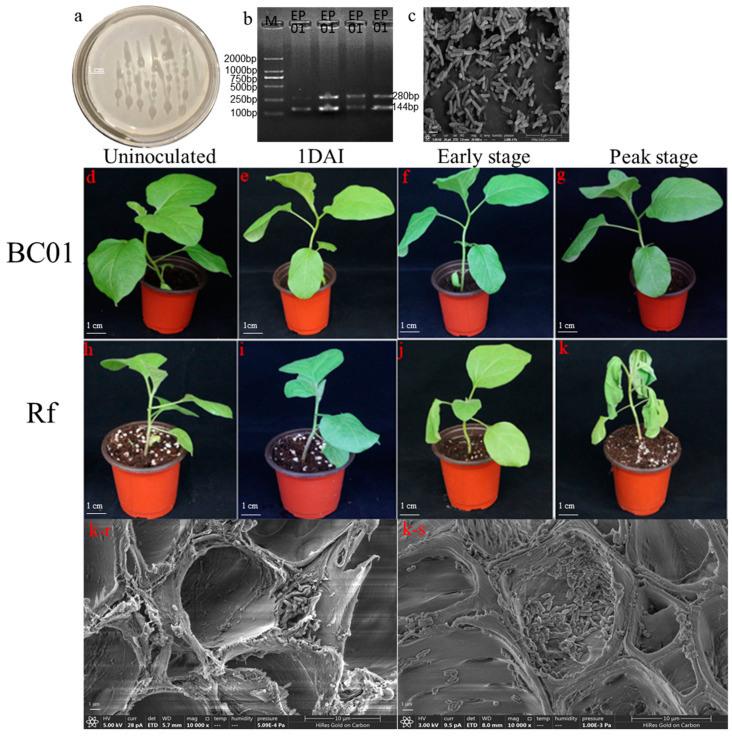
Identification of *Ralstonia solanacearum*, plant phenotype, and histological observation. (**a**) *R. solanacearum* in NA medium, (**b**) Specific and phylotype DNA fragments of *R. solanacearum* in agarose electrophoresis. (**c**) *R. solanacearum* under scanning electron microscopy (SEM). (**d**–**k**) Plant phenotype at four stages: uninoculated, 1 day post-inoculation, early disease onset, and peak disease onset in BC01 (**d**–**g**) and Rf (**h**–**k**). (**k-r**,**k-s**) *R. solanacearum* invaded and propagated in the roots and stems of plants with three or more withered leaves in Rf under SEM.

*R. solanacearum* EP01 was white, milky, and liquid in the nutrient agar (NA) medium (Figure 1a). The bacterial solution was selected for molecular amplification and SEM observation. The EP01 showed a 280 bp specific band and a 144 bp phylotype I band (Figure 1b). EP01 was short rod under SEM (Figure 1c). After inoculation with *R. solanacearum*, Rf gradually presented with leaf wilting, while BC01 had no symptom of wilting (Figure 1d–k). At the peak of disease onset, Rf had three or more withered leaves. Furthermore, *R. solanacearum* was found in higher abundance in the stem than in the root in Rf (Figure 1k-r,k-s).

### 2.2. Analysis of Transcriptome Data

The root and stem of BC01 and Rf were sequenced at the uninoculated, 1 day post-inoculation, early disease onset, and peak disease onset stages. After performing low-quality sequencing of the original reads, the results showed that all samples gained a high-quality base of more than 7.0 GB. The percentage of Q30 bases was >92.21%. The content of GC was >41.99%, indicating that the transcriptional data were reliable and could be used for subsequent analysis (Additional file S1: Table S1).

The comparison analysis of the reference genome showed that all samples were comparable to the reference genome. The average number of effective sequences ranged from 47,464,161 to 66,897,973. The comparison rate of a single location was >91.86%, while that of multiple locations was <3.25%. According to the comparison results of total mapped reads, the exon, intron, and intergenic regions of the reference genome were 84.99%, 7.65% to 10.79%, and 3.79% to 5.45%, respectively (Additional file S1: Table S2).

### 2.3. Module Division and Soft Threshold Selection

**Figure 2:**
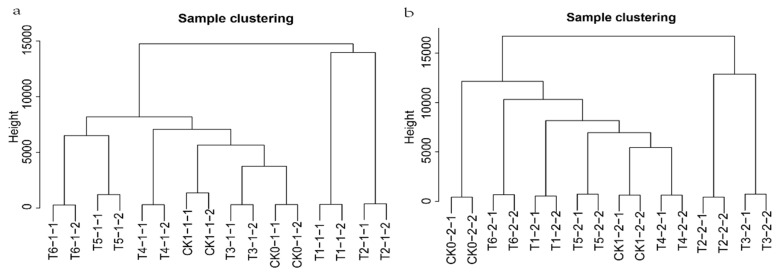
Sampling repeatability. (**a**,**b**) Hierarchical clustering tree showed that there are no outliers in root samples and in stem samples according to the expression of all genes.

For the WGCNA clustered genes with similar expression patterns, we divided them into different modules and analysed the relationship between modules and specific traits or phenotypes. This method has been widely used in research of phenotypic traits and gene association analysis. We selected the gene sets with FPKM average expression ≥2. Low-quality data were removed for WGCNA analysis (root: 21,245 genes, stem: 20,941 genes) (Additional file S1: Tables S3 and S4). To avoid the influence of outlier samples, a hierarchical clustering tree was constructed based on the expression of all genes (Figure 2a,b). The repeatability of the sample relationship was good, which was conducive for follow-up analysis. 

**Figure 3:**
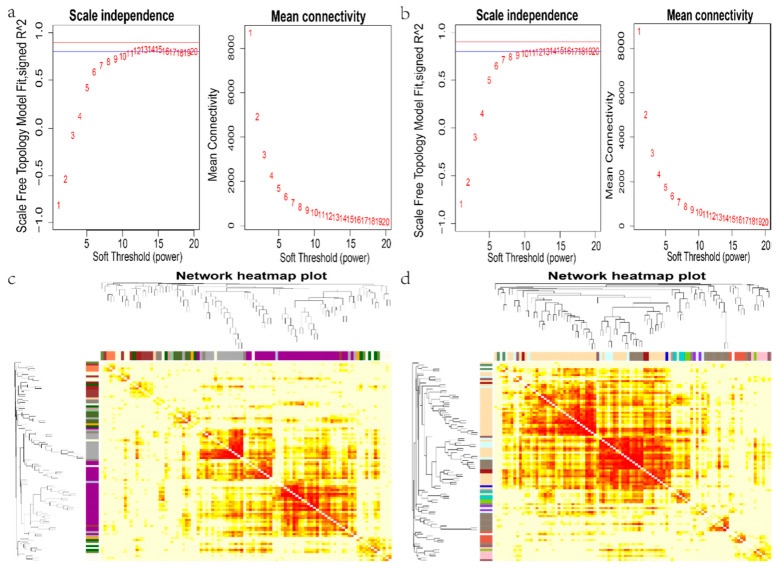
Co-expression network analysis identifying gene modules underlying eggplant resistant to *Ralstonia solanacearum* infection. (**a**,**b**) Soft threshold dividing co-expressed genes into different modules in the root and stem. (**c**,**d**) Topological overlap matrix (TOM) heatmap showing the correlation among gene module memberships in the root and stem.

Based on the identified genes in WGCNA, the formula Sij = |cor(xi,xj)| determined the correlation between the two genes; aij = power(Sij,β) = |Sij|^β^ then calculated the scale-free result. We integrated the numbers, entered the platform stage and the average connectivity, and selected a soft threshold, β = 12, in the root and stem tissues to construct the network (Figure 3a,b). To explain the relationship between modules and genes, the topological overlap matrix (TOM) was established by calculating the adjacency matrix and correlation matrix of gene expression profile (Figure 3c,d). 

### 2.4. Interest Module Mining

**Figure 4:**
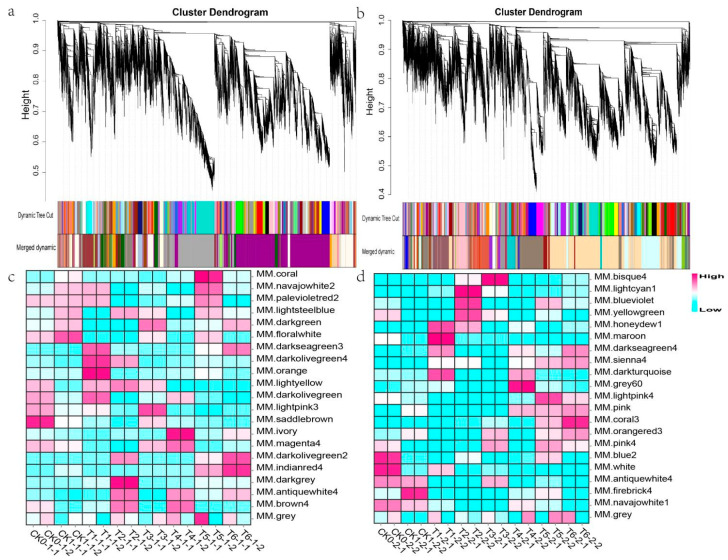
Identification of gene modules related to eggplant resistance to *Ralstonia solanacearum* infection. (**a**,**b**) Hierarchical cluster trees showing co-expression modules identified using WGCNA of the differentially expressed genes in the root (**a**) and stem (**b**). The grey module contained genes that could not be classified into any module. (**c**,**d**) Heatmaps showing module–sample expression pattern in the root (**c**) and stem (**d**). Magenta to cyan represents high to low expression.

To divide different modules, the cluster tree was constructed according to the correlation of gene expression by the dynamic cutting method. The genes with similar expression patterns were classified into the same module when different modules (root: 21, stem: 21) were generated (Figure 4a,b). Each module had a specific expression pattern. To determine the most interesting modules, we drew a heatmap of sample expression patterns by conducting a correlation and cluster analysis between samples and modules. Meanwhile, the expression pattern of module genes in each sample was displayed by module eigenvalues (Figure 4c,d). The module eigenvalue was equivalent to the weighted comprehensive value of all gene expression in the module, which reflected the comprehensive expression level of all genes in the module in each sample. We aimed to find genes related to resistance to bacterial wilt. Except for indianard4 in the root and coral3 in the stem, the expression level trend of other modules showed irregular changes with BC01 and Rf. When BC01 and Rf were not inoculated, the expression levels of indianred4 in the root and coral3 in the stem were low, and Rf was lower than BC01. After inoculation, Rf had a low expression level, while BC01 expression level gradually changed from low to high. At the peak of the disease, the two modules had the highest expression with BC01 (Figure 4c,d). Therefore, in each of the 21 modules of the roots and stems, a module of interest was mined to further explore the candidate pathways and hub genes related to disease resistance.

### 2.5. Enrichment Analysis of GO and KEGG Pathways

**Figure 5:**
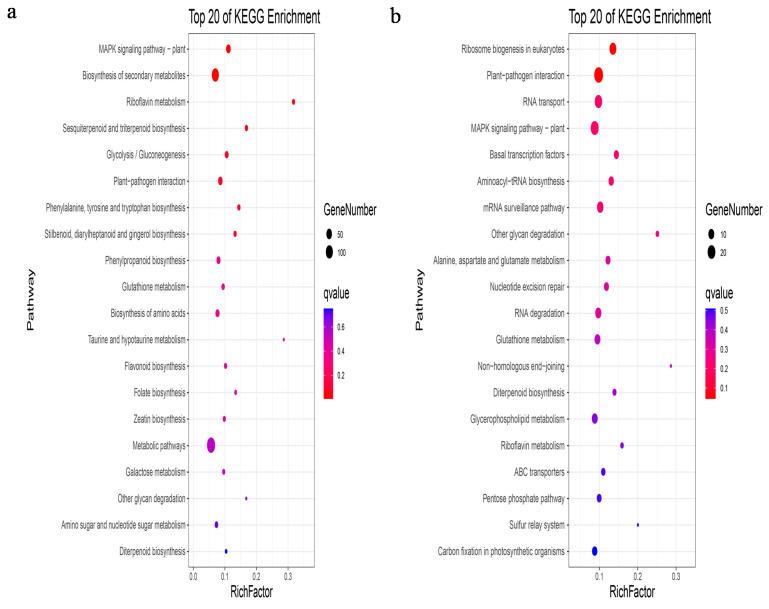
Path analysis of co-expressing modules of interest. (**a**,**b**) Top 20 KEGG-enriched pathways of indianred4 in the root and coral3 in the stem. The columns and rows represent pathways and richfactor. The richfactor refers to the ratio of the number of genes located in the KEGG pathway in the DEGs to the total number of genes located in the KEGG pathway, for all annotated genes. The round size shows the number of genes; colour bar shows the significance with qvalue. The qvalue is the false discovery rate (FDR)-corrected pvalue; redder colour indicates greater significance.

To further explore the function of the module of interest, the genes of this module were mapped to each term of the GO database for GO function enrichment analysis. The selected module genes in roots and stems could be enriched into three kinds of functions: cell component, molecular function, and biological process (Additional file S2: Figures S1 and S2). Furthermore, the pathways of the selected modules were anchored, and a hypergeometric test was applied to find the pathways that were significantly enriched in the selected modules compared with the whole genome background in the KEGG database. The indianred4 module genes in roots was significantly related to the MAPK signalling pathway–plant, biosynthesis of secondary metabolites, riboflavin metabolism, sesquiterpenoid and triterpenoid biosynthesis, glycolysis/gluconeogenesis, plant–pathogen interaction, phenylalanine, tyrosine and tryptophan biosynthesis, stilbenoid, diarylheptanoid and gingerol biosynthesis, phenylpropanoid biosynthesis, glutathione metabolism, biosynthesis of amino acids, taurine and hypotaurine metabolism, and flavonoid biosynthesis. The coral3 module gene in the stem was significantly associated with the following pathways: ribosome biogenesis in eukaryotes, plant–pathogen interaction, RNA transport, MAPK signalling pathway–plant, basal transcription factors, aminoacyl-tRNA biosynthesis, mRNA surveillance pathway, other glycan degradation, alanine, aspartate and glutamate metabolism, nucleotide excision repair, RNA degradation, glutathione metabolism, and non-homologous end-joining (Figure 5a,b).

### 2.6. Mining Hub Genes Related to Disease Resistance

**Figure 6:**
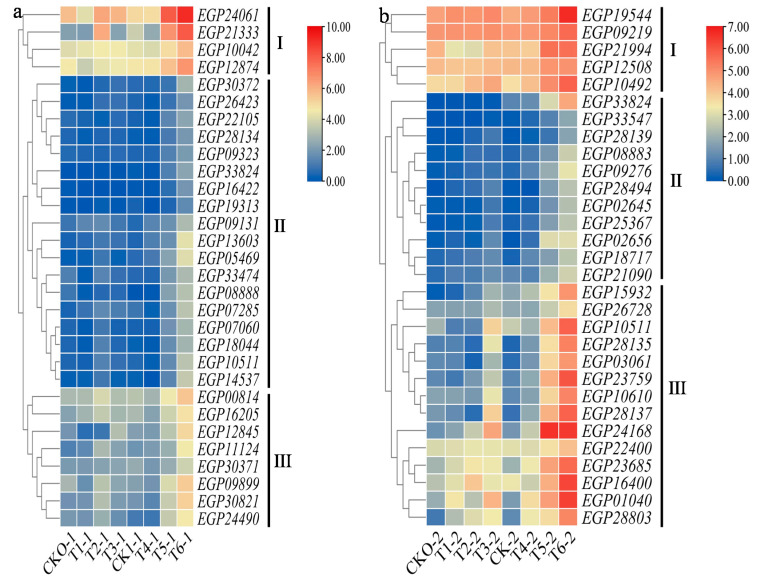
Heatmaps showing the top 30 expression pattern related to eggplant resistance to *Ralstonia solanacearum* infection in the root (**a**) and stem (**b**). The columns and rows in the heatmap represent genes and samples. The colour bars explain the scale used to indicate gene expression level; this scale represents the logarithm of FPKM values of gene expression, red to blue represent high to low expression.

The hub gene was further mined from the module of interest in the root and stem through All.kWithin value (Additional file S1: Tables S5 and S6). The higher the absolute value of All.kWithin, the more important the gene was. There were 1636 genes in the root of the indianred4 module and 1302 genes in the stem of the coral3 module. The expression changes of the each top 30 hub genes (presented in the genome) in roots and stems were observed, and the genes with similar expression patterns were classified into three categories by heatmap (Figure 6a,b). The characteristics of each class follow: Class I gene expression at the same high level on BC01 and Rf, and class II gene expression at the same low level on BC01 and Rf. The expression levels of class III were different, but the trend was that the expression levels were at the same level in the uninoculated and 1 d post-inoculation, and there was no significant change. In the early disease onset, and peak disease onset, the expression levels were high on BC01 and low on Rf. The expression levels of all selected hub genes in BC01 were higher than those in Rf at the early disease onset, and peak disease onset. We noticed that these selected hub genes have a large number of receptor kinase genes and are mainly distributed in class II (root: *EGP28134*, *EGP33824*, *EGP09323*, *EGP26423*, *EGP18044*, stem: *EGP02645*, *EGP08883*, *EGP09276*, *EGP33824*, *EGP25367*, *EGP33547*, *EGP28139*), the root EGP12874 was distributed in class I, and the stem *EGP10610* and *EGP28137* were distributed in class III. The root *EGP00814* of NBS-LRR resistance protein are in class III. At the same time, we also observed the expression of all transcription factors in the module (58 in the root and 38 in the stem) using the heatmap (Additional file S2: Figures S3 and S4). The expression of most transcription factors on BC01 and Rf was not significantly different, but we still found that WRKY, BHLH, NAC family transcription factors, root *EGP08888* (*BHLH119*), *EGP22612* (*WRKY70*), *EGP10141* (*WRKY40*), *EGP19681* (*WRKY40*), and *EGP28029* (*WRKY51*), stem *EGP18717* (*WRKY53*), *EGP26518* (*BHLH14*). *EGP21008* (*WRKY53*), *EGP29469* (*NAC022*), and *EGP02966* (*BHLH111*) had high expression in BC01 and low expression in Rf.

### 2.7. Expression of Selected Genes

According to KEGG pathway enrichment analysis or the top 30 hub genes, combined with NR annotation, 14 genes (8 from root and 6 from stem) were selected for qPCR (Additional file S1: Table S7).

**Figure 7:**
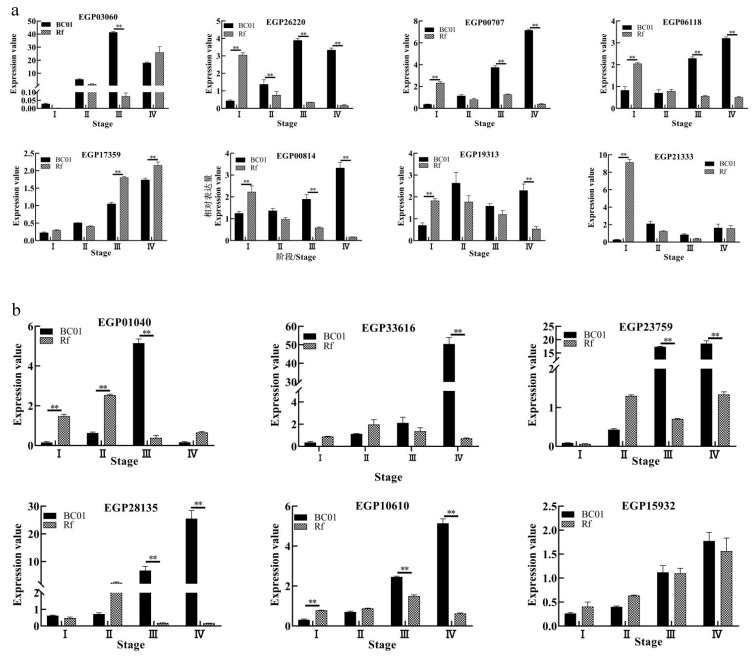
Expression analysis of candidate genes screened under WGCNA. (**a**) A total of 8 candidate genes were selected from root transcriptome results and verified by qPCR in root tissues. (**b**) A total of 6 candidate genes were selected from stem transcriptome results and verified by qPCR in stem tissues. The bar plots and error bars represented averages and standard errors for three repeated samples under *t*-test. I, uninoculated; II, 1 day post-inoculation; III, early disease onset; IV, peak of disease onset. Note: ** indicates significance at the 0.01 level.

The comparison rate between the expression levels of 14 candidate genes and FPKM of transcriptome data was 78%. The trend was basically the same (Additional file S2: Figures S5 and S6). Among the eight candidate genes in the root (Figure 7a), the expression of the four most interesting genes in the root, *EGP26220*, *EGP00707*, *EGP06118*, and *EGP00814*, increased gradually in BC01 but decreased gradually in the root of Rf. The expressions of the four genes in the root of BC01 were significantly higher than those in the root of Rf in the early and peak stages of the disease. The expression levels of *EGP26220*, *EGP00707*, and *EGP06118* involved in riboflavin synthesis in BC01 roots were 3 to 17 times those of Rf (Figure 7a). Among the six candidate genes in the stem (Figure 7b), the expression of the three most interesting genes in the stem, *EGP23759*, *EGP28135*, and *EGP10610*, showed a gradual upward trend in the stem of BC01, and a gradual upward or first upward and then downward trend in the stem of Rf. When *EGP23759*, *EGP28135*, and *EGP10610* were expressed in the early stage and peak stage of the disease, the expression level in stem of BC01 was significantly higher than that of Rf (Figure 7b).

### 2.8. Network Regulation of Interest Genes

**Figure 8:**
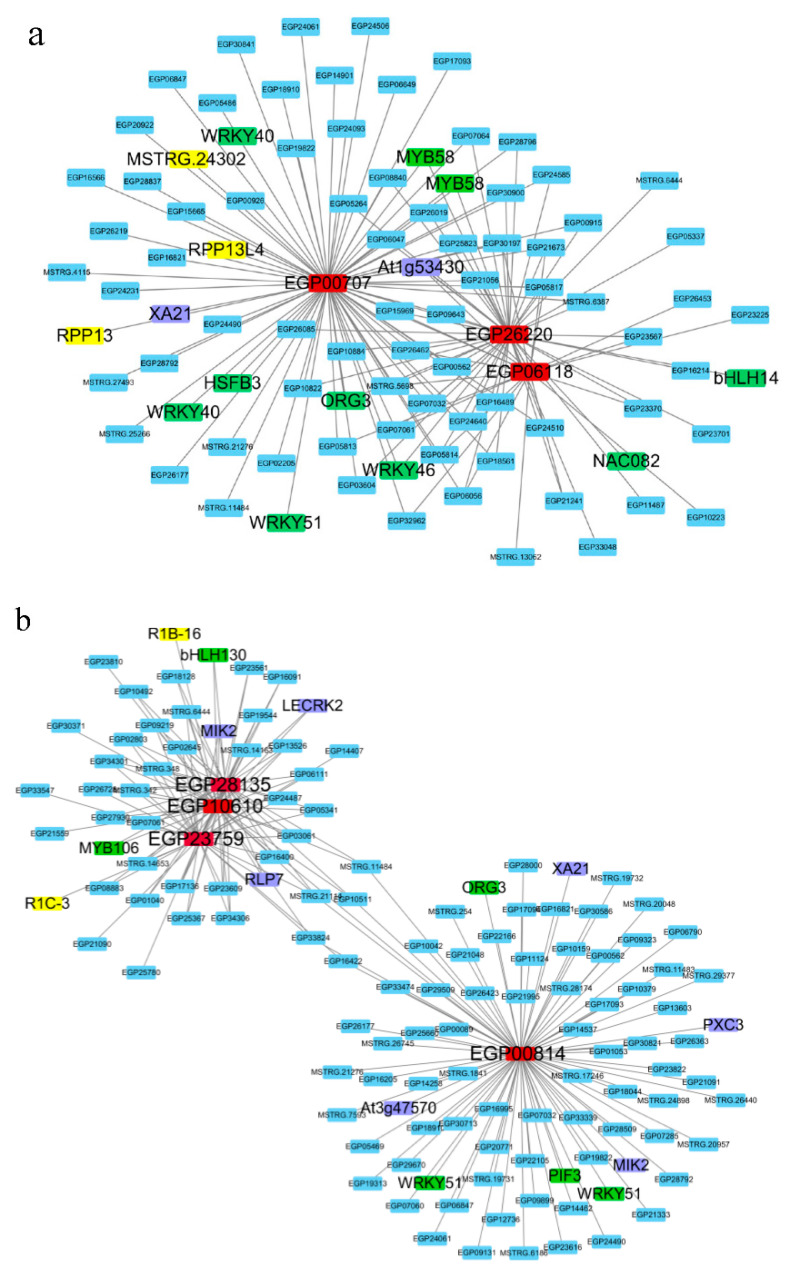
Network regulation of the genes of interest (**a**) Genes interacting with riboflavin pathway-related genes *EGP26220*, *EGP00707*, and *EGP06118*. (**b**) Genes interacting with hub genes *EGP00814*, *EGP23759*, *EGP28135*, and *EGP10610*. Red shows genes of interest, green shows TFs, violet shows disease resistance genes of receptor kinase family, and yellow shows other disease resistance genes.

Based on the above qPCR results, we noticed that riboflavin pathway-related genes *EGP26220*, *EGP00707*, and *EGP06118*, and the four hub genes *EGP00814*, *EGP23759*, *EGP28135*, and *EGP10610*, were well-represented. To further understand the possible regulatory mechanism of these genes, the regulatory network was constructed. At the root, there were 1636 genes interacting with riboflavin pathway-related genes *EGP26220*, *EGP00707*, and *EGP06118* (Additional file S1: Table S8). A total of 90 interaction genes were mapped by weight ≥0.25, and eight transcription factors were found: *MYB58*, *WRKY40*, *WRKY46*, *WRKY51*, *HSFB3*, *ORG3*, *NAC082*, and *bHLH14*, and five resistance genes *MSTRG.24302* (unknown symbol), *XA21*, *At1g53430*, *RPP13*, and *RPP13L4* (Figure 8a). There were 1778 genes interacting with hub genes *EGP00814*, *EGP23759*, *EGP28135*, and *EGP10610* (Additional file S1: Table S9). A total of 131 interaction genes were mapped by weight ≥0.35, and five transcription factors were found: *WRKY51*, *ORG3*, *PIF3*, *bHLH130* and *MYB106*, and eight resistance genes: *MIK2*, *PXC3*, *At3g47570*, *XA21*, *R1B-16*, *RLP7*, *LECRK2*, and *R1C-3*. At the same time, there is an interaction among *EGP00814* and *EGP28135*, *EGP23759*, and *EGP10610* (Figure 8b). 

### 2.9. Functional Verification of SmRPP13L4 Gene

**Figure 9:**
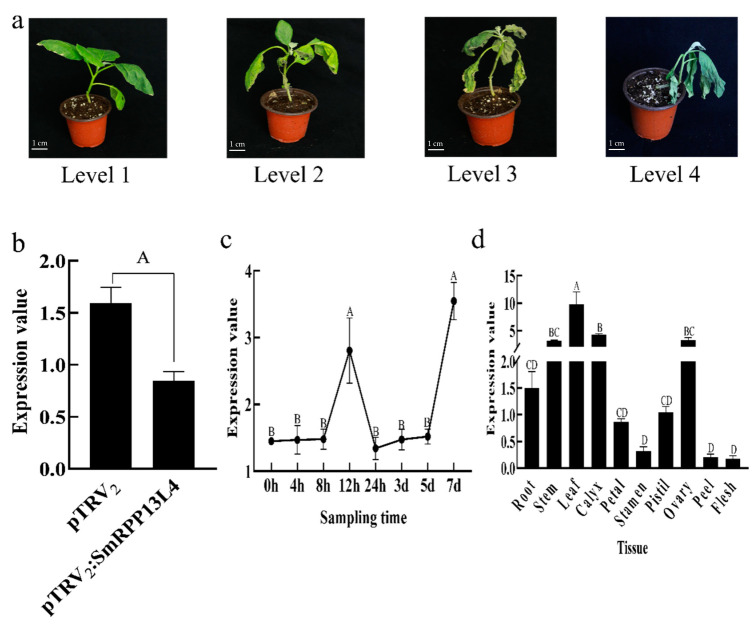

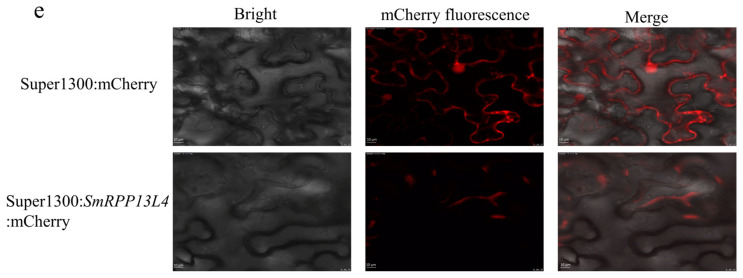
Functional verification of *SmRPP13L4* gene. (**a**) The disease grade aboveground in BC01 after silencing *SmRPP13L4* gene. (**b**) *SmRPP13L4* gene expression after silencing *SmRPP13L4* in stem. The bar plots and error bars represent averages and standard errors for three repeated samples under *t*-test. (**c**) The expression of *SmRPP13L4* gene in BC01 root at different times of *R. solanacearum* infection. The points and error bars represent averages and standard errors for three repeated samples under Duncan’s test. (**d**) Expression of *SmRPP13L4* gene in different tissues of BC01. The bar plots and error bars represent averages and standard errors for three repeated samples under Duncan’s test. (**e**) Subcellular localization of *SmRPP13L4* gene. Note: capital letters show significance at the 0.01 level.

According to the above results, the expression of *EGP00814* (symbol: RPP13L4, namely *SmRPP13L4*) gene was significantly different from BC01 and Rf. It is speculated that it plays an important role in eggplant resistance to bacterial wilt. Virus-induced gene silencing of the *SmRPP13L4* gene was carried out. The disease phenotype of plants 28 days after inoculation with *R. solanacearum* (Table 1, Figure 9a) showed that the disease index of plants with *SmRPP13L4* gene silencing increased by 13.13, and the incidence increased by 37.5%, and the expression of *SmRPP13L4* in gene silencing plants was downregulated by 50% (Figure 9b), indicating that the disease resistance of BC01 was weakened by silencing *SmRPP13L4* gene. With the inoculation of *R. solanacearum* in BC01, the root tissues at different times were taken. *SmRPP13L4* gene showed a trend of first increase and then decrease from 0 to 24 h, and reached a peak after 12 h, then decreased, and then increased from 24 h to 7 d (Figure 9c). In BC01 normal plants, the *SmRPP13L4* gene had high expression in leaf, stem, calyx and ovary, and low expression in peel and flesh (Figure 9d). The *SmRPP13L4* gene position was located in the cell membrane (Figure 9e).

## 3. Discussion

### 3.1. Module of Interest Played a Considerable Role in Eggplant Bacterial Wilt-Resistance

When pathogens invade plants, the immune system actively recognises and responds to pathogens by producing corresponding regulatory pathways to resist the infection of pathogens. The modules of interest selected in this study were expressed significantly in the early disease onset and peak disease onset after inoculating with the *R. solanacearum*. KEGG pathways in the root and stem co-enriched MAPK signalling pathway, plant–pathogen interaction, and glutathione metabolism. Previous studies showed that MAPK was a highly conserved signalling pathway that possessed the ability of stress acceptance and transmission and played a key role in plant resistance to phytoplasma attack. MAPK cascade was involved in the signal transduction of various defence responses, including the biosynthesis of signal transduction of plant stress/defence hormones, production of reactive oxygen species (ROS), stomatal closure, activation of defence genes, phytoalexin biosynthesis, cell wall strengthening, and hypersensitivity (HR) cell death [21,22,23]. A series of *FLS2* genes were found in the MAPK signalling pathway and plant–plant pathogen interaction. *FlS2* was a PRR with good characteristics, which could recognise a conserved 22 amino acid antigen from bacterial flagellin (flg22) and played a role in disease resistance [24,25]. In glutathione metabolism, transcriptomics and proteomics studies showed that some *GST* genes were upregulated specifically in infected plants. The accumulation of *GST* protein in infected plants was regulated by binding with glutathione (*GSH*) to detoxify toxic lipid hydrogen peroxide, reduce oxidative stress, and participate in hormone transport [26,27]. In addition, the riboflavin metabolism pathway was enriched in the root, which showed a highly significant correlation. Riboflavin is a type of water-soluble vitamin B (VB_2_) that can enhance the stress and disease resistance of crops. Previous studies showed that riboflavin stimulated the production of H_2_O_2_, upregulated a series of defence-related genes, synthesised callose in stomatal cells, participated in octadecanoic acid pathway, stimulated lipoxygenase (*LOX*), and strengthened lignification and other mechanisms in regulating plant disease resistance [28,29]. In addition, the present study showed that the expression levels of *EGP26220* (encodes 6,7-dimethyl-8-ribityllumazine synthase), *EGP00707* (encodes riboflavin synthase), and *EGP06118* (encodes riboflavin biosynthesis protein Riba1) in the BC01 roots were significantly higher than those of Rf. This result suggested that the module of interest has a great impact on the resistance of eggplant to bacterial wilt. The information such as pathways and genes in the module lays a foundation for further research on the resistance of eggplant to bacterial wilt.

### 3.2. Candidate Bacterial Wilt-Resistance Hub Gene Mining

Hub genes are of great significance in interpreting plant phenotypes. A large number of receptor kinases are enriched in the top 30 hub genes. Receptor kinases located on the cell membrane can bind to receptor proteins to identify pathogens, mediate the first immune defence line PTI reaction in plants, and effectively block the invasion and reproduction of pathogens [30,31,32,33]. Sometimes, receptor kinases also affect the R gene-mediated disease resistance pathway. WRKY, BHLH, NAC, and other transcription factors are involved in plant disease resistance mechanisms. In Solanaceae crops, *CaWRKY40*, *CaWRKY6*, *CaWRKY58*, *CaBHLH94*, and *StNACb4* transcription factors were identified to be involved in the regulation of bacterial wilt [34,35,36]. Additionally, we observed the change rules of expression levels of all transcription factors in the selected disease resistance module, and identified *WRKY40*, *WRKY51*, *WRKY53*, *WRKY70*, *BHLH14*, *BHLH111*, *BHLH119*, and *NAC022*. The expression levels of these transcription factors were significantly different. Combined with qPCR results, among the 14 disease resistance candidate genes, four significant hub genes were screened: *EGP10610*, *EGP00814*, *EGP23759*, and *EGP28135*. *EGP00814* (namely *RPP13L4*) belonging to the NBS-LRR gene, interacted with *WRKY51*, which was identified as belonging to this family; it contains CC, NBS, and LRR domains and plays an important role in defence against downy mildew of barley and brown planthopper of peanut [37,38,39]. Additionally, *RPP13*-lk3 presented stress resistance. Previous studies have shown that *RPP13*-lk3 is a new adenylate cyclase (AC), which could catalyse ATP to produce 3′,5′-cAMP and regulate heat tolerance [40]. *EGP23759*, *EGP28135*, and *EGP10610* interacted with *BHLH130* and *MYB106*. *EGP28135* (namely *MIK2*) and *EGP10610* (namely *SOBIR1*) belonged to receptor kinase genes, *MIK2* maintained cell wall integrity and *SOBIR1* could cause plant cell death; these two genes activated the pathogen-resistance responses [41,42]. *EGP23759* encodes WAS protein family homolog DDB_G0292878-like isoform X1. The four hub genes interacted with five genes of the receptor kinase family. Combined with phenotype, BC01 had no wilt in the early stage and peak stage of disease, and Rf gradually showed leaf wilt. The expression levels of this hub gene in BC01 were significantly higher than those of Rf. In summary, these results provide a theoretical reference for the candidate genes of eggplant bacterial wilt-resistance. At the same time, the hub gene that belongs to the module and was not analysed has important scientific significance for eggplant bacterial wilt-resistance.

### 3.3. SmRPP13L4 Regulated Eggplant Bacterial Wilt-Resistance

Previous studies have shown that root resistance is an integral part of plant resistance to bacterial wilt. *R. solanacearum* infection could enter the root of host through physical trauma or natural openings. Generally, it occupied the intercellular space of root cortex within 24 h, and the resistance of plant root was activated accordingly. Later, *R. solanacearum* continued to infect the intercellular space of root vascular parenchyma, and the cells of degraded parenchyma invaded xylem vessels. *R. solanacearum* in susceptible plants was easy to proliferate in large quantities and spread upward along the stem, resulting in blockage of xylem and wilting of plants [43,44,45,46]. QPCR results showed that the *SmRPP13L4* gene showed a continuous upward trend before 12 h within 24 h after inoculation with *R. solanacearum* in the roots of BC01, and maintained a continuous upward trend from 24 h to 7 d. After *SmRPP13L4* gene was silenced, and the gene expression was downregulated, and the plant resistance to bacterial wilt was decreased. At the same time, the *SmRPP13L4* gene was located in the cell membrane, it had tissue specificity, the expression levels of leaf, stem, calyx and ovary were high, and the expression levels of peel and flesh were low. Therefore, *SmRPP13L4* is a membrane protein mainly distributed in leaf, stem, calyx and ovary, which positively regulates the resistance of eggplant to bacterial wilt.

## 4. Materials and Methods

### 4.1. Materials

Two kinds of eggplant lines, BC01 (bacterial wilt-resistant eggplant) and Rf (bacterial wilt-sensitive eggplant), were collected from the germplasm resources nursery of the vegetable base in the teaching and scientific research department of the Agricultural College of Guangxi University (22.5100 N, 108.1725 E), China. BC01 is a highly resistant cultivar of purple oval fruit eggplant, and Rf is a highly susceptible cultivar of purple long fruit eggplant [47]. Seeds of BC01 and Rf were soaked in 1 g/L gibberellin for 3 h. They were dried and directly seeded in a 72-hole tray with peat, vermiculite, and perlite at a ratio of 3:1:1. They were then placed at a constant room temperature (28 °C) until germination. When they grew six leaves, they were moved to a 32-hole tray and were then inoculated.

### 4.2. Identification and Inoculation of Ralstonia solanacearum

*R. solanacearum* (EP01) [48] was provided by the plant pathology laboratory of Guangxi University. Its specificity and phylotypes were identified by phylotype-specific multiplex PCR using a set of phylotype-specific primers and species-specific primers (Additional file S1: Table S10) and 2 × A8 FastHiFi PCR Mastermix amplification (Aidlab, Beijing, China). The settings of the PCR cycle were as follows: pre-denaturation at 95 °C for 5 min, followed by 35 cycles at 94 °C for 15 s, 59 °C for 30 s, and 72 °C for 30 s, with a final extension at 72 °C for 10 min. The final result showed the 280 bp specific reference fragment, and 144 bp phylotype I fragment, 372 bp phylotype II fragment, 91 bp phylotype III fragment, or 213 bp phylotype IV fragment. It was cultured on NA medium for 48 h and was shaken in a liquid NA medium for 10 h. The final concentration of *R. solanacearum* was 5 × 10^8^ cfu/mL. Seedlings were inoculated with *R. solanacearum* using the root perfusion method. Each plant was inoculated with 30 mL. They were then placed at a constant room temperature room (28 °C) after inoculation. The disease was counted from 3 to 28 d after inoculation of *R. solanacearum*, and the phenotype was investigated according to the Winstead grading standard to evaluate the disease resistance level, 0 level show no obvious symptoms, 1 level show 1 leaf wilting, 2 level show 2–3 leaves wilting, 3 level show more than 4 leaves wilting except the terminal bud, 4 level show the whole plant wilting or death. Disease index = ∑(disease grade × disease grade plant number)/(highest disease grade × survey plant number) × 100.

### 4.3. Scanning Electron Microscopy 

The 1 to 2 mm main root and stem tissues were taken, fixed with 2.5% glutaraldehyde for 48 h, washed with 0.01 mol/L PBS three times, dehydrated with ethanol gradient containing 70%, 85%, and 95% for 10 min each and with 100% ethanol for 15 min, and dried at 37 °C in an oven. Thereafter, two to three samples were placed on every sample table of ion sputtering gold and observed by SEM. Each sample was randomly observed in 10 views at 5000× magnification and photographed at 10,000× magnification. 

The bacterial solution of *R. solanacearum* was cultured in a liquid NA medium to OD_600_ = 0.8 and centrifuged at room temperature for 2 min. The supernatant was discarded to collect the precipitated bacteria, which were fixed with 2.5% glutaraldehyde for 2 h. The steps were the same as above.

### 4.4. RNA-Seq and Analysis

The roots and stems of BC01 and Rf were sequenced at four stages, namely the uninoculated, 1 day post-inoculation, early disease onset, and peak disease onset stages. After inoculation 3–5 d into the early disease onset, Rf showed one withered leaf, while BC01 showed no symptom of wilting. After inoculation 6–7 d into the peak disease onset, Rf showed three or more withered leaves, whereas BC01 showed no symptom of wilting. Samples for each of these stages were taken on the same day. This was repeated twice, and a total of 32 samples were obtained. CK0 corresponded to Rf not inoculated with *R. solanacearum*, wherein sampling was done on the day of inoculation. T1, T2, and T3 corresponded to Rf 1 day post-inoculation, early disease onset, and peak disease onset stages, respectively. CK1 corresponded to BC01 not inoculated with *R. solanacearum*, wherein sampling was done on the day of inoculation. T4, T5, and T6 corresponded to BC01 at 1 day post-inoculation, early disease onset, and peak disease onset stages. Notably, −1 corresponded to roots, while −2 corresponded to stems. RNA was extracted with the Omega plant RNA kit (Omega Bio-tek, Norcross, GA, USA). It was used to construct an RNA library and to sequence on the Illumina NovaSeq 6000 instrument sequencing platform (Genedenovo Biotechnology Co., Ltd., Guangzhou, China). Fastp (v0.18.0) [49] was used to check the quality of raw reads and to select clean reads. Bowtie2 (v2.2.8) [50] was used to compare the short reads with the ribosome database of the species and to remove the reads of the upper ribosome without mismatch. The remaining unmapped reads were used for subsequent transcriptome analysis. HISAT 2 (v2.4) [51] was used to compare the sequence with the reference genome; the parameter was set as ‘-rna-strandness RF’. StringTie (v1.3.1) [52] was used to reconstruct the transcription to find new genes. RSEM (v1.2.19) [53] was used to calculate gene expression. Sample relation utilisation used R (http://www.r-project.org/, accessed on 25 March 2020) to perform a principal component analysis (PCA) with a goal of dimension reduction. The Pearson test was used for the correlation analysis. The DESeq (v1.24.0) [54] was used to analyse gene differential expression (FDR ≤ 0.05 and log2|fold change| ≥ 1). All expressed genes were transferred to the GO database (http://www.geneontology.org/, accessed on 19 April 2020). The main biological functions of genes were determined by the GO annotation. The main biochemical metabolic pathway and signal transduction pathway of genes were analysed by using the KEGG database. The differential genes were analysed by WGCNA. The WGCNA analysis was carried out by WGCNA (v1.47) [15] package in R, and the soft threshold was determined by scale-free network principle. The genes with similar expression patterns were clustered and divided into modules. The gene-module correlation was analysed by Pearson correlation, and the expression pattern of the module and the sample was calculated. The intra-module connectivity (All.kWithin) of each gene was calculated to determine the Hub gene. The parameters used in the software were default except in the need for changes.

### 4.5. Virus-Induced Gene Silencing (VIGS)

The VIGS vector was constructed by double enzyme digestion. The EcoRI and BamHI restriction sites on the multi-cloning sites of pTRV_2_ vector were selected, and the 300 bp specific fragment of target gene silencing was determined by using the VIGS Tool of Solanaceae Genome Website (https://solgenomics.net/, accessed on 25 April 2021). The primers were designed and protective bases, EcoRI and BamHI endonuclease sequences (Takara, Dalian, China), were added to the 5′ end of the forward and reverse primers (Additional file S1: Table S11). The cDNA of eggplant BC01 leaves was used as a template to amplify and obtain a specific product of 300 bp fragment, and fused with pTRV_2_ vector. The resultant pTRV_2_-gene construct and empty vector (pTRV_1_, PTRV_2_) were introduced into agrobacterium GV3101 (HuaYueYang, Beijing, China) by the freeze–thaw method.

*Agrobacterium tumefaciens* containing pTRV_2_-gene, pTRV_2_, and pTRV_1_ were added to LB liquid medium containing kanamycin (Kana, 100 mg/mL) and rifampicin (Rif, 50 mg/mL) at a ratio of 1:100 at 28 °C and 180 rpm for 48 h. The shaken bacterial solution was diluted to fresh LB liquid medium (containing 100 mg/mL Kana, 50 mg/mL Rif, 10 mM MES and 20 μm acetosyringone) at 1:50, and shaken overnight at 28 °C and 180 rpm. The cells were collected by centrifugation at 5000 rpm for 5 min, and the supernatant was discarded. The cells were resuspended with MMA (100 mL distilled water containing 10 mM MgCl_2_, 10 mM MES and 200 μm acetosyringone), and the OD_600_ was about 1.0. The suspension containing pTRV_1_ was mixed in equal volume with the suspension containing pTRV_2_ and pTRV_2_-recombinant vectors, respectively. Then, the suspension was incubated in dark at 25 °C for 4 h. Finally, the mixture of empty pTRV_2_ and pTRV_1_ was used as the control. The infection was carried out in four true leaves of BC01 plants. The inoculation was artificially inoculated by root-injured irrigation and cotyledon injection. After inoculated, the plants were cultured in darkness at 16 °C for 1 d, then placed in 25 °C, 16 h light/8 h darkness for 10 d, and inoculated with *R. solanacearum*. Each treatment was inoculated with 40 plants. 

### 4.6. Subcellular Localization

The overexpression vector was constructed using the Seamless Assembly Cloning Kit (Clone Smarter, Houston, TX, USA). The plasmid super-1300 was digested by Xbal (Takara, Dalian, China) and KpnI (Takara, Dalian, China). The overexpressed genes were cloned on BC01 using specific primers (Additional file S1: Table S11). The fragment containing the same 18 bp homologous sequence as the Xbal and kpnI sites of the vector at the 5′/3′ site was the full length CDS that removed the termination codon, and was fused with the super1300 vector. The resultant super-genes–mCherry construct and empty vector were introduced into agrobacterium GV3101 (HuaYueYang, Beijing, China) by the freeze–thaw method. We selected a single *Agrobacterium* colony and shook it in 5 mL LB liquid medium (containing Kana and Rif) at 28 °C and 180 rpm. The OD_600_ was about 1.0. We then discarded the supernatant after centrifugation at 8000 rpm for 2 min, added 5 mL MMA buffer to suspend gently, adjusted the inoculation buffer to an OD_600_ about 0.5, and let them stand at room temperature in the dark for 3 h after inoculation.

A disposable syringe was used to suck 1 mL mixed bacterial solution. The needle was gently taken to prick a small hole on *N. benthamiana* leaves, the inoculated tobacco was cultured at 25 °C for 24 h, and then cultured for 2–3 d, and observed under laser confocal microscope (Leica, Wetzlar, Germany). 

### 4.7. Quantitative Reverse Transcription (qPCR) Analysis

RNA was extracted using the Eastep^TM^ Super Total RNA Extraction kit (Promega, Shanghai, China). cDNA was synthesised using the Primescript™ RT Kit. qPCR was performed using gene-specific primers (Additional file S1: Table S12) and the TB Green Premix Ex TaqII Kit (Takara, Dalian, China) following the manufacturer’s instructions. The settings of the qPCR cycle follow: pre-denaturation at 95 °C for 30 s, denaturation at 95 °C for 5 s, and annealing at 60 °C for 34 s. Actin and L25 [55] were used as the internal reference of eggplant and tobacco. The expression of all samples was 3 repeats. The expression data of related genes were analysed by 2^−ΔΔCt^.

### 4.8. Statistical Analysis

SPSS (v18.0) software was used to analyse the significance by *t*-test (two groups) or Duncan’s test (three groups or more). Graph Pad Prism 8 (v8.0.2) and TBtools (v1.098669) [56] were used to plot graphs. Cytoscape (v3.7.2) was used to draw networks. 

## Figures and Tables

**Table 1:** Statistics of bacterial wilt after silencing SmRPP13L4 gene.

Gene Silencing	Number of Disease Grade Plants					Disease Index	Incidence Rate %
	Level 0	Level 1	Level 2	Level 3	Level 4		
pTRV_2_ (CK)	35	0	2	2	1	8.75	12.5
pTRV_2_:*SmRPP13L4*	20	11	4	4	1	21.88	50

## Data Availability

The raw sequencing data generated from this study have been deposited in NCBI SRA (https://submit.ncbi.nlm.nih.gov/sra/, accessed on 11 May 2021) under the accession number PRJNA728497 (https://dataview.ncbi.nlm.nih.gov/object/PRJNA728497?reviewer=1irfe20b1oo1smifbq4jhdd8cc, accessed on 11 May 2021).

## Supplementary Materials

All date are available online at https://www.mdpi.com/article/10.3390/ijms222413279/s1**.**
