# Supplementary figures and images for: Weighted Gene Co-Expression Analysis Network-Based Analysis on the Candidate Pathways and Hub Genes in Eggplant Bacterial Wilt-Resistance: A Plant Research Study
Source: Int J Mol Sci. 2021 Dec 10;22(24):13279. doi: 10.3390/ijms222413279 (PMC8706084; doi:10.3390/ijms222413279)

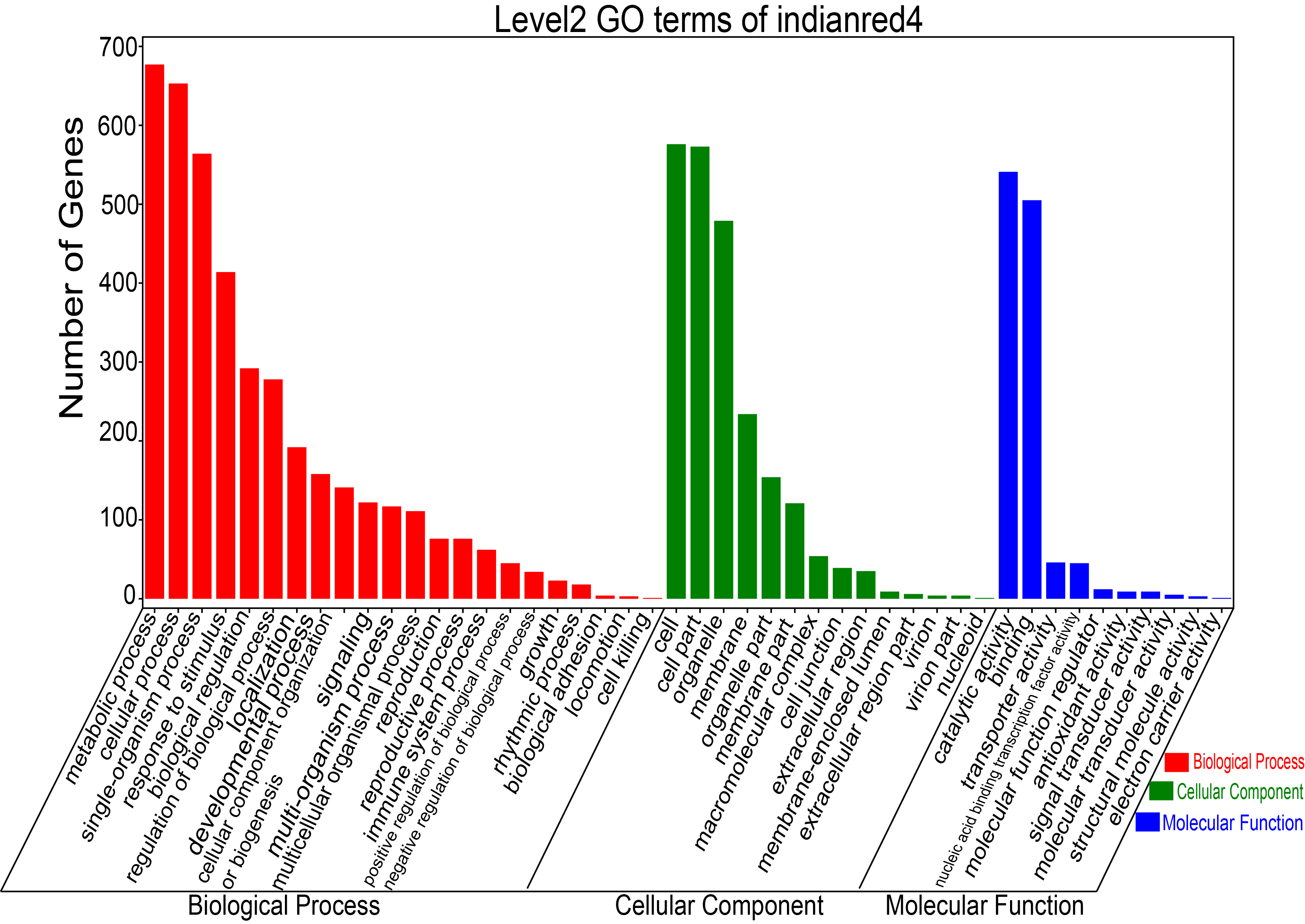

Supplement: Supplementary file 1 [file ijms-22-13279-s001.zip › Additonal file S2/Figure S1. GO terms of indianred4 in the root.png]

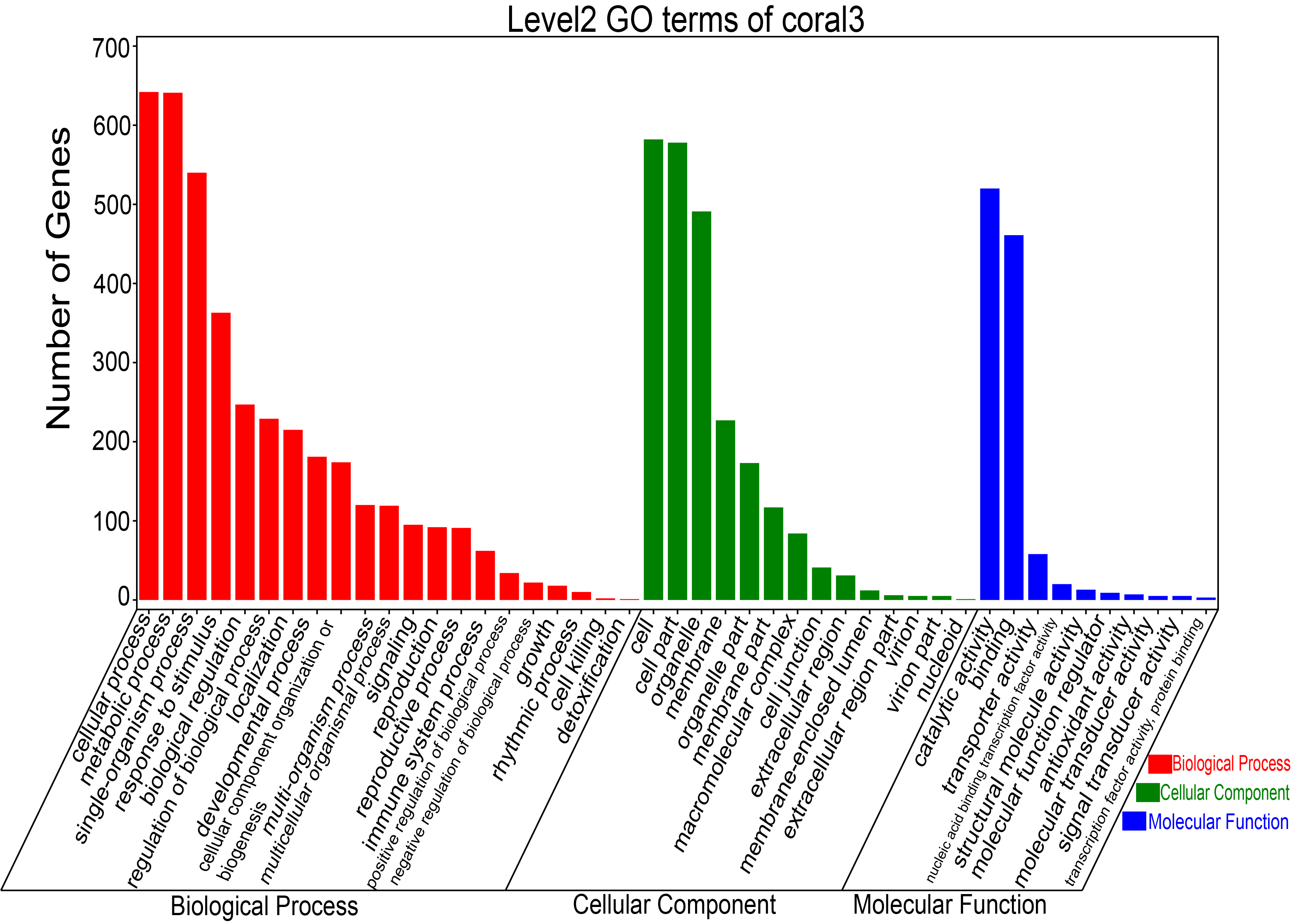

Supplement: Supplementary file 1 [file ijms-22-13279-s001.zip › Additonal file S2/Figure S2. GO terms of coral3 in the stem.png]

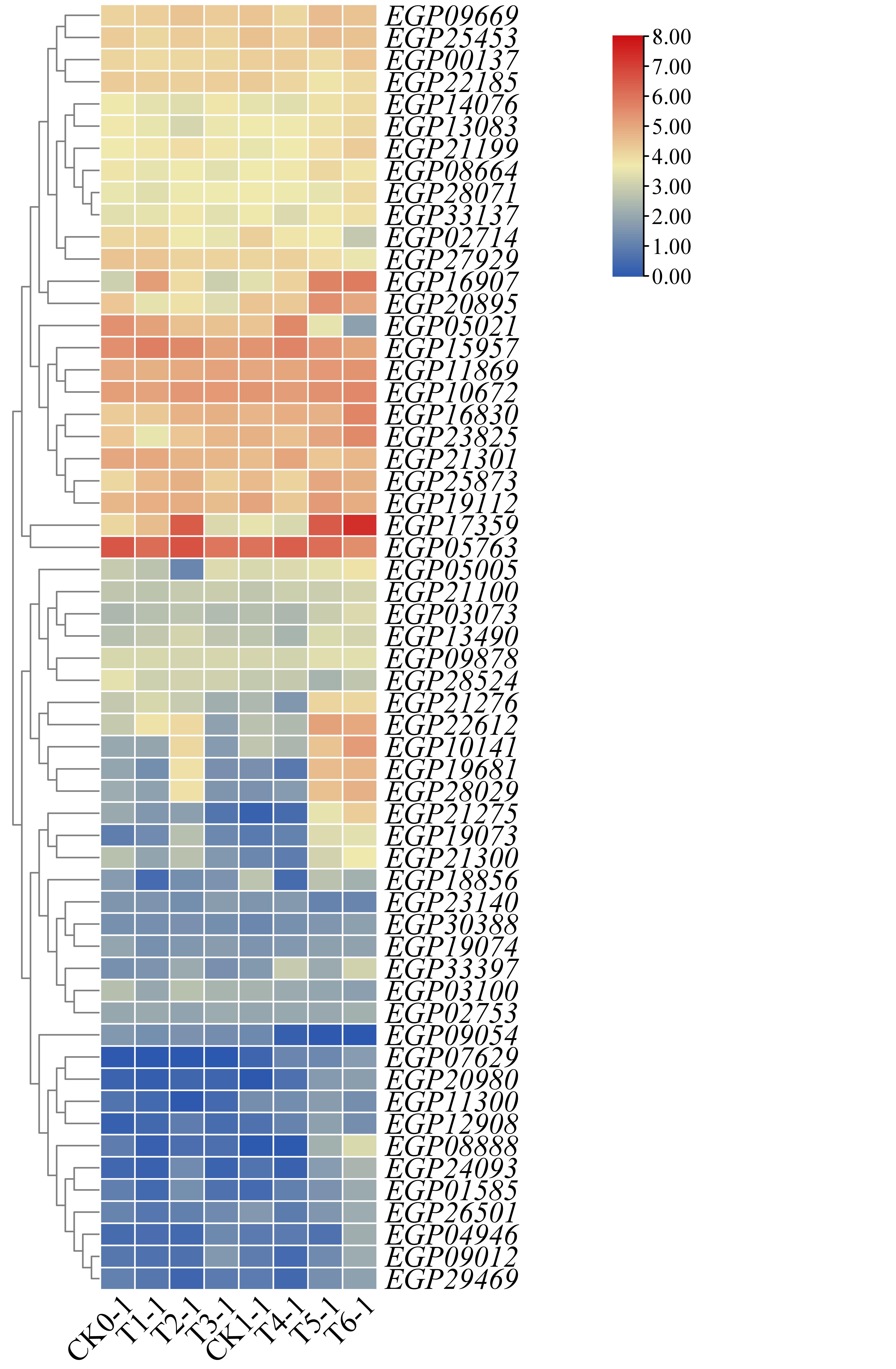

Supplement: Supplementary file 1 [file ijms-22-13279-s001.zip › Additonal file S2/Figure S3. Heatmap showed the expression of root transcription factor.jpg]

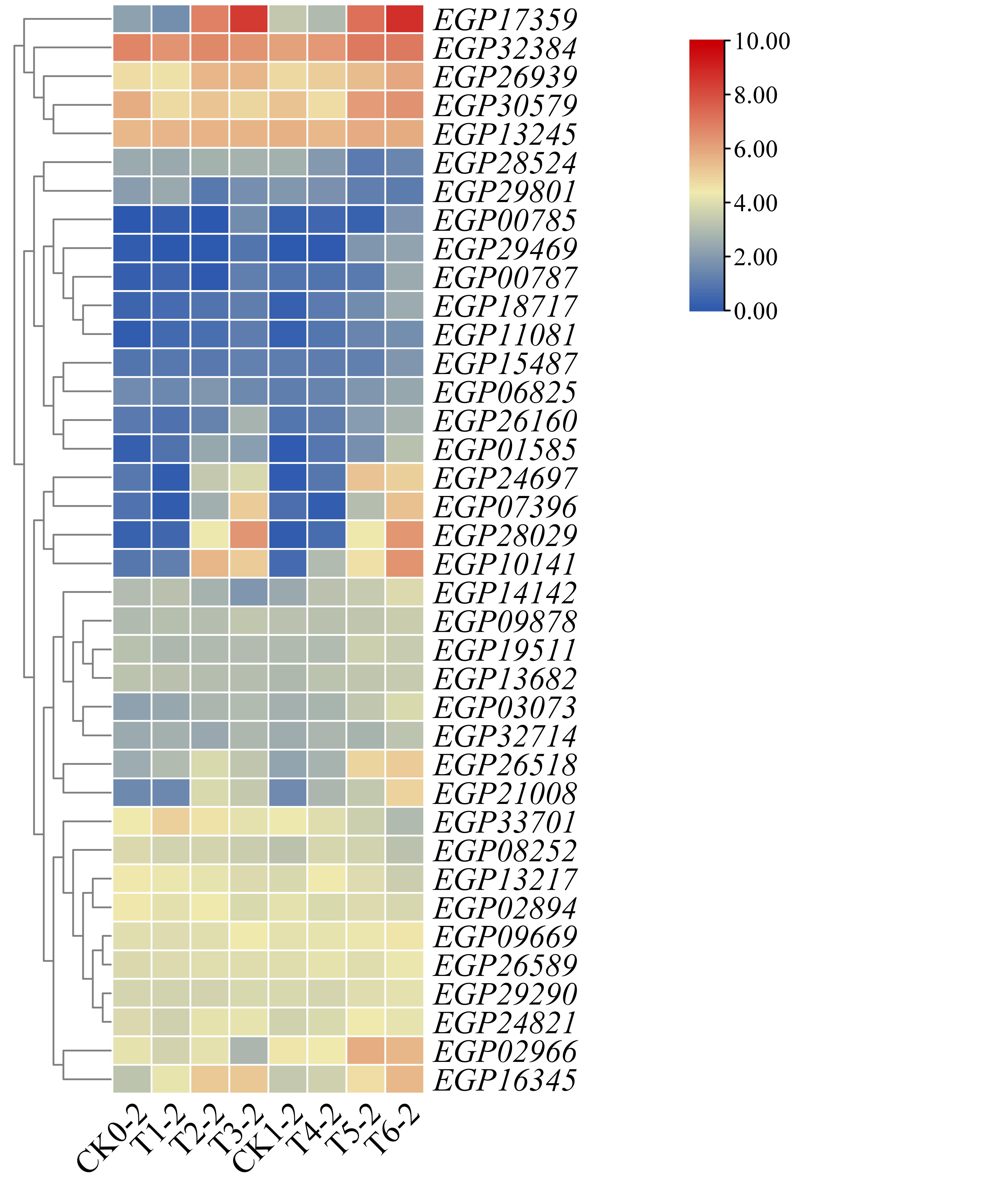

Supplement: Supplementary file 1 [file ijms-22-13279-s001.zip › Additonal file S2/Figure S4. Heatmap showed the expression of stem transcription factor.jpg]

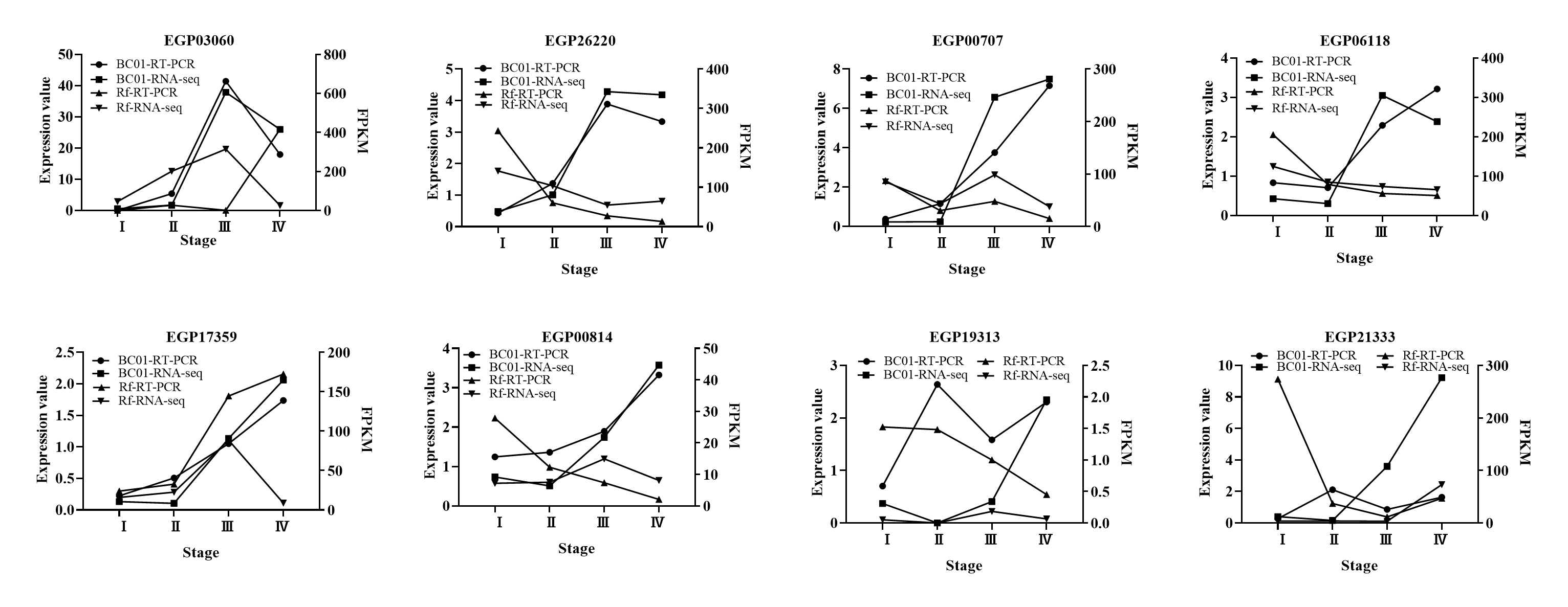

Supplement: Supplementary file 1 [file ijms-22-13279-s001.zip › Additonal file S2/Figure S5. Comparison of root candidate genes by qRT-PCR and RNA-seq.png]

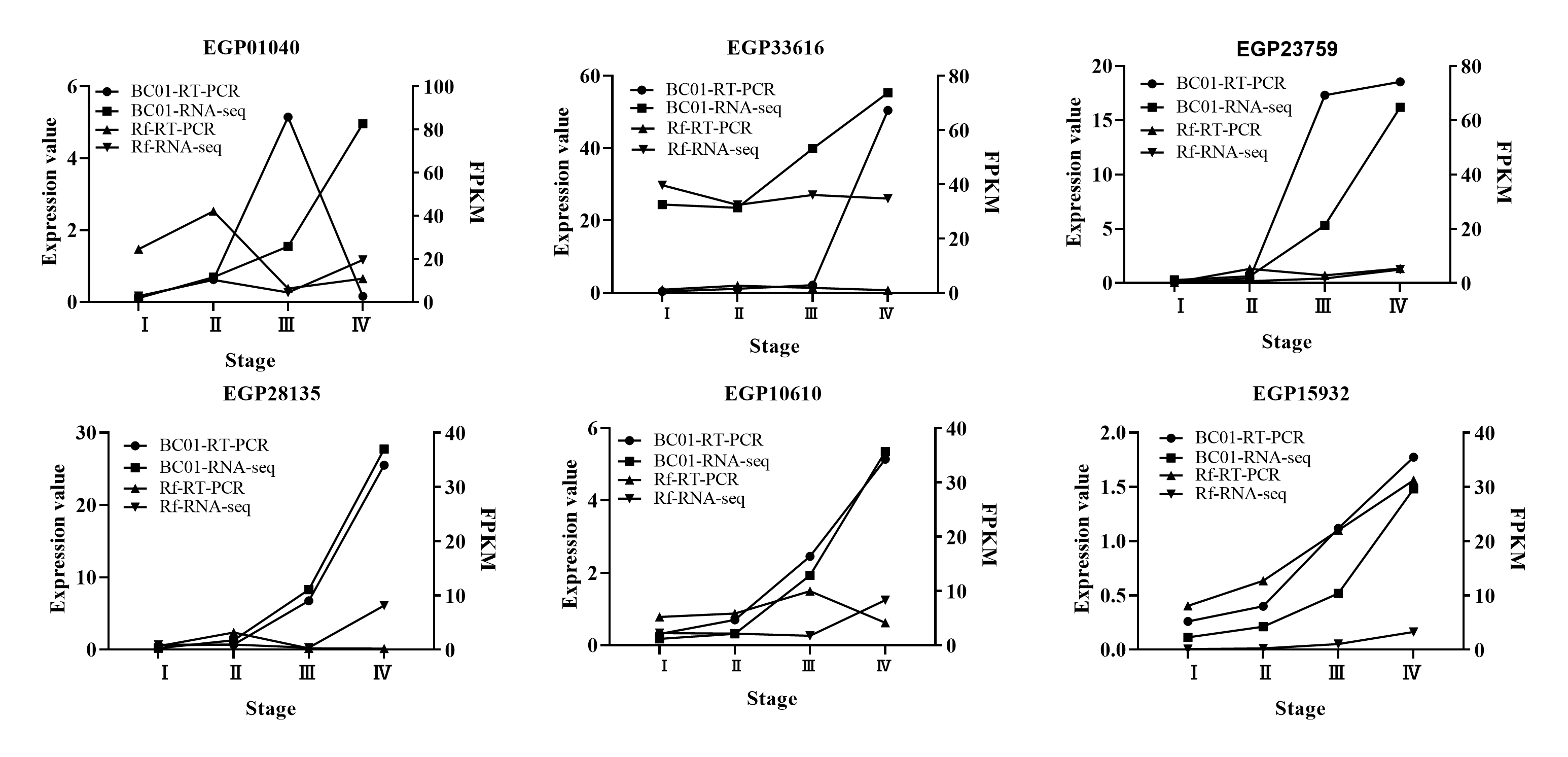

Supplement: Supplementary file 1 [file ijms-22-13279-s001.zip › Additonal file S2/Figure S6. Comparison of stem candidate genes by qRT-PCR and RNA-seq.png]
